# COVID term: a bilingual terminology for COVID-19

**DOI:** 10.1186/s12911-021-01593-9

**Published:** 2021-08-03

**Authors:** Hetong Ma, Liu Shen, Haixia Sun, Zidu Xu, Li Hou, Sizhu Wu, An Fang, Jiao Li, Qing Qian

**Affiliations:** Institute of Medical Information/Library, Chinese Academy of Medical Sciences/Peking Union Medical College, Beijing, China

**Keywords:** COVID-19, Terminology system, Bilingual, Medical terminology

## Abstract

**Background:**

The coronavirus disease (COVID-19), a pneumonia caused by severe acute respiratory syndrome coronavirus 2 (SARS-CoV-2) has shown its destructiveness with more than one million confirmed cases and dozens of thousands of death, which is highly contagious and still spreading globally. World-wide studies have been conducted aiming to understand the COVID-19 mechanism, transmission, clinical features, etc. A cross-language terminology of COVID-19 is essential for improving knowledge sharing and scientific discovery dissemination.

**Methods:**

We developed a bilingual terminology of COVID-19 named COVID Term with mapping Chinese and English terms. The terminology was constructed as follows: (1) Classification schema design; (2) Concept representation model building; (3) Term source selection and term extraction; (4) Hierarchical structure construction; (5) Quality control (6) Web service. We built open access for the terminology, providing search, browse, and download services.

**Results:**

The proposed COVID Term include 10 categories: disease, anatomic site, clinical manifestation, demographic and socioeconomic characteristics, living organism, qualifiers, psychological assistance, medical equipment, instruments and materials, epidemic prevention and control, diagnosis and treatment technique respectively. In total, COVID Terms covered 464 concepts with 724 Chinese terms and 887 English terms. All terms are openly available online (COVID Term URL: http://covidterm.imicams.ac.cn).

**Conclusions:**

COVID Term is a bilingual terminology focused on COVID-19, the epidemic pneumonia with a high risk of infection around the world. It will provide updated bilingual terms of the disease to help health providers and medical professionals retrieve and exchange information and knowledge in multiple languages. COVID Term was released in machine-readable formats (e.g., XML and JSON), which would contribute to the information retrieval, machine translation and advanced intelligent techniques application.

## Background

The coronavirus disease (COVID-19), a type of pneumonia caused by severe acute respiratory syndrome coronavirus 2(SARS-CoV-2) was known in December 2019 [1]. The patients showed typical symptoms such as fever, cough, fatigue, and even respiratory failure [2, 3] especially for high-risk people with chronic disease or decreased immunity [4]. With extremely high infectiousness, COVID-19 confirmed cases have accumulated to 83,597 in China till April 13, 2020 [5]. The Chinese government took effective control measures including scaling up diagnostic testing, isolating suspected cases, classifying, tracking, and managing contacts of confirmed cases and restricting mobility [6], limiting events and gatherings, and calling for universal wearing of masks. COVID-19 experienced an outbreak worldwide, till April 13, 2020, the confirmed cases in the whole world have been 1,773,084 with 111,652 deaths [5].

Medical studies have been conducted to investigate COVID-19 globally. Clinical findings were analyzed via different test methodologies [7], and in diverse groups (e.g. children, pregnant patients, regular patients, critically ill patients [8–12]). Modelling studies were applied in different areas [13–16] and towards disparate directions, e.g. control strategy interventions, dynamic transmission, etc. [6, 15–17]. Clinical case studies were conducted in a specific region, or a specific domain e.g. risk factors [18, 19]. Virus-related features and distribution analyses [20] were also investigated. Semantic and geographical analysis towards COVID-19 trials were completed to reveal the trends [21]. Liu et.al. completed an ontology towards coronavirus named Coronavirus Infectious Disease Ontology (CIDO) and targeting at drug identification and repurposing [22].Plenty of techniques (e.g. artificial intelligence, virtual reality, internet of things (IoT), internet of medical things (IoMT), etc.) were applied in COVID-19 related clinical and practical research [23–26]. With the production of mass exponential data, knowledge graphs covering genes, pathways, and expression, etc. were constructed and various data processing methods were applied for drug repurposing and treatment including deep learning, graph representation learning, neural networks, etc. [27, 28].

As COVID is causing increasingly great loss, researchers in multiple areas especially in medical field strongly demand for latest scientific publishment such as published literatures. The literatures are conveyed in different languages. However, it is simpler for people to search resources with language they are used to [29], thus the requirement for retrieval in different languages has largely risen. Knowledge dissemination can be promoted through automatic machine technique, for instance a cross-lingual professional system. While more structured machine-readable data should be fed to the intelligent techniques such as deep learning model to realize further data mining and ontology construction, and cross-lingual terminology is necessary to be created. Additionally, parallel machine-readable corpus would contribute to more applications (e.g. machine translation). In this study, we constructed a bilingual COVID terminology named COVID Term aiming to accelerate the spread of COVID knowledge across different countries, support the bilingual retrieval requirement towards COVID-19, and provide bilingual machine-readable data for intelligent techniques.

## Methods

To enable the reuse of terminology data for ontology and knowledge graph construction, we referred to the top-down strategy for building domain specific ontologies [30, 31], and authoritative terminology system ICD, SNOMED CT for terminology criteria establishment [32, 33]. The designed workflow included six steps, respectively (1) Classification schema design (2) Concept representation model building (3) Term source selection and term extraction (4) Hierarchical structure construction(5) Quality control (6) Web service. All the editing part was performed on TBench, a work platform for cross-lingual terminology system editing and maintenance [34].

### Classification schema design

The purpose of the classification schema design was to define a certain scope of the whole terminology, focusing on the important branches that need to be included. The classification schema design should take multiple dimensions of research in COVID-19 into consideration, e.g. the research direction of epidemiology and a specific disease, the data structure design, etc. Specifically, in terms of epidemiology, 8 categories were proposed including person status, affected group, disease distribution, disease spreading, incidence, occurrence; etiology, and the disease understanding [35]. Still, other information categories were necessary to be mentioned (e.g. diagnosis method and treatment technique) and the terminology construction shared quite different emphasis compared to epidemiological concern, more requirements have to be considered especially when establishing a indexing-, annotation-, and retrieval-oriented terminology system. We also referred to other structure of terminology system, e.g. SNOMED CT where body structure, clinical finding, environment or geographical location, event, observable entity, organism, specimen, substance, etc. were incorporated. Since this category system was designed mainly for clinical use and general medical field, it might not be appropriate for a specific disease (e.g. COVID-19). To get more comprehensive perspectives for clinical and data researchers, we consulted experts in medical informatics and achieved agreements. We then developed 10 classification schema for the first level top nodes involving disease, anatomic site, clinical manifestation, demographic and socioeconomic characteristics, living organism, qualifiers, psychological assistance, medical equipment, instruments and materials, epidemic prevention and control, diagnosis and treatment technique.

### Concept representation model building

**Fig. 1:**
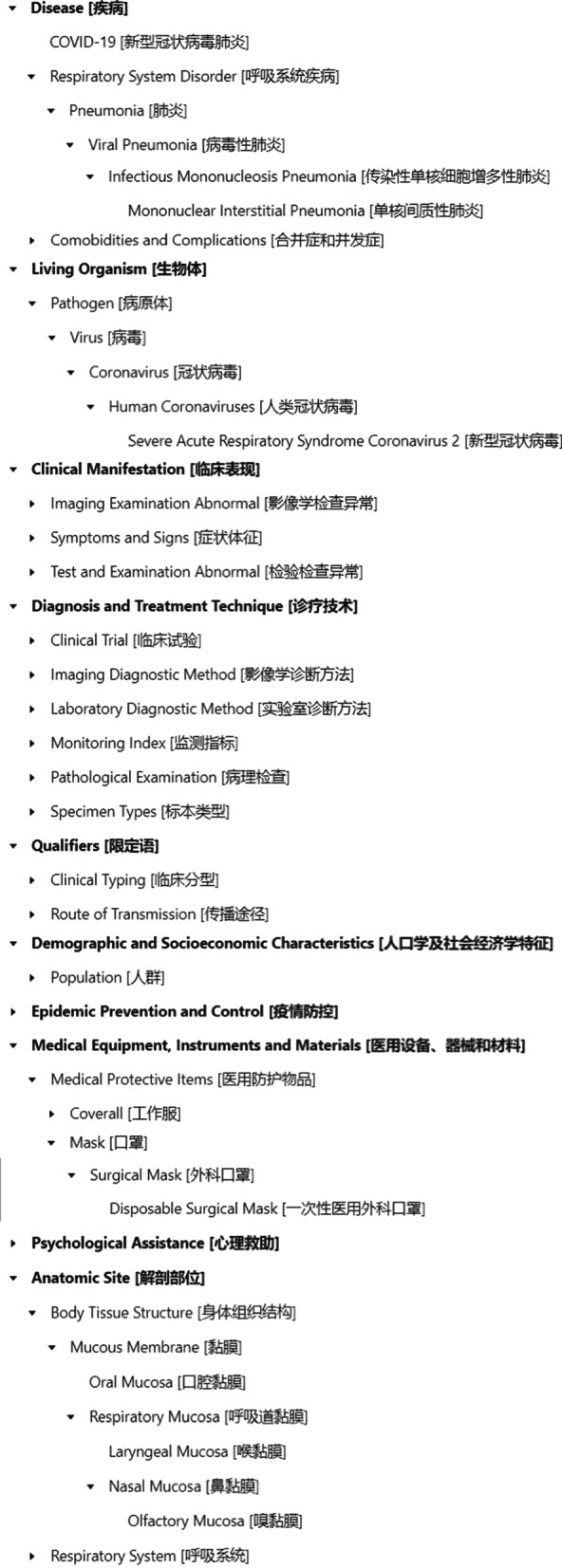
The concept representation model

Referring to SKOS Simple Knowledge Organization System [36], we designed a concept representation model where all the terms were organized on the basis of its core concept. As shown in the concept representation model (Fig. 1), each core concept was assigned one particular concept ID and three elements, i.e. definition, term, and semantic type. The semantic type represents the most similar category and meaning of one concept, it is an efficient way to retrieve concepts and terms that have a certain semantic type. Each term was designed with attributes of term ID, lexical value (term content), term source, preferred term flag (whether this term is preferred term or synonym), and language. Semantic type ID, its lexical value and language information were introduced in semantic type. Definition involved lexical value, definition source and language information. In terms of relationships, each concept but leaf concept has sub concept of another concept. Each concept not in the top category is the sub concept of another concept. Each concept has its term, definition and semantic type. Each term is the term of one concept. Each definition is the definition of one concept. Each semantic type is the semantic type of one concept.

### Term source selection and term extraction

A bilingual terminology system towards a worldwide emergency disease was supposed to be correct, authoritative and highly correlated to the theme, where exact bilingual concepts, semantic types, etc. should be demonstrated. Therefore, the information resources we took were limited to authority publishment from the situation report or document of World Health Organization, journal articles such as preprint, open access, etc., nationwide regulation, policy document, professional textbooks, etc. Bilingual terms were mostly extracted from bilingual WHO documents, textbooks, and related papers. Definitions were located from textbooks and related papers under most conditions.

### Hierarchical structure construction

**Fig. 2:**
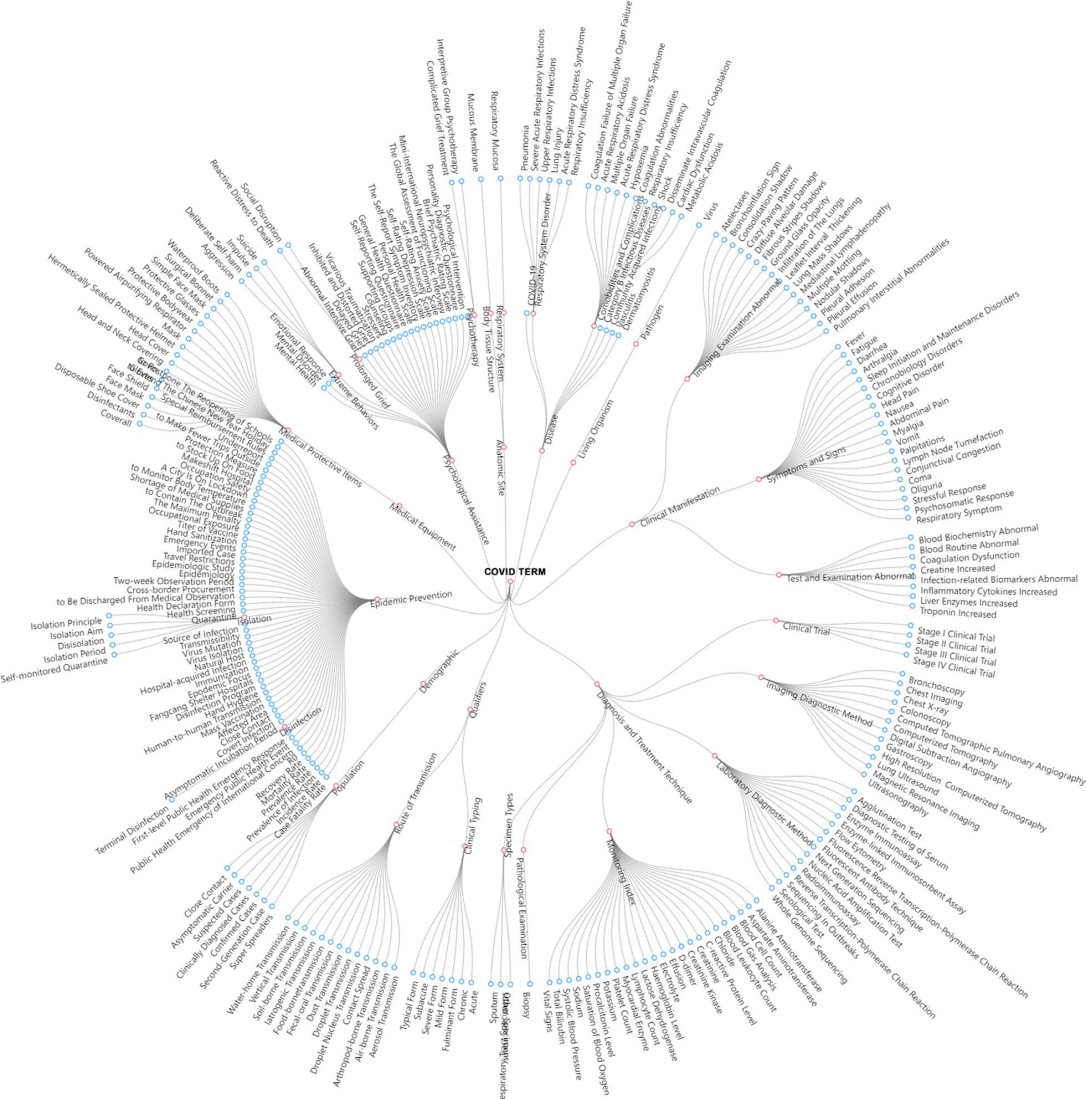
Hierarchical structure of COVID Term

We adopted top-down and bottom-up synthesis approach to formulate the final hierarchical structures. On the one hand, related clinical classification architectures were identified and reused in associated documentation, literatures, textbooks (e.g. textbooks in epidemiology, virology, and preclinical medicine), etc. On the other hand, measures were taken for terms extracted from literatures and other resources, i.e. synthesis from bottom up. Agile model was adopted during the procedure, i.e. adjusting the structure by adding, altering or deleting specific substructures when necessary. The finalized hierarchical structure (Fig. 2) was reviewed and assessed by one expert on clinical medicine and one two professionals on medical informatics.

### Relationship and property development

Relationship and property of each concept were developed in this step. Each concept was assigned with properties including concept ID, term ID, bilingual semantic type, Chinese preferred term, and English preferred term as the obligatory items, and Chinese synonym, English synonym, bilingual definition, definition source as alternative items. We combined terms with same meanings as one concept with different synonyms; integrated terms within the same classification as one subset with various concepts. The editing date and time were automatically generated in the system. Among these properties, concept ID and term ID could be directly linked to other systems through automatic mapping; each definition was required with a source for users to look up to. Synonyms were not a prerequisite element but more synonyms would help with the search scope and term location.

### Quality control

To guarantee the correctness of the terminology, we performed quality control after each round editing and before each version update. After editing in each round, two examiners with professional background and related practice experience were invited to validate the accuracy of the terminology. A third party with clinical experts would be involved when disagreement was reached. Before each releasing round, we performed quality control via cross assessment, i.e. automatic checking and expert review. The former was responsible for repeated terms detection (i.e. repeated terms with different concept identification), language detection (i.e. English terms marked as Chinese or vice versa), abnormal character detection (i.e. term that cannot be read by machine), closed hierarchical relationship detection (e.g. whether there is circularity in a hierarchical tree), hierarchical depth detection (whether a term is too deep for users to browse), spelling detection (whether there is questionable spelling). The expert review covered classification checking (whether the classification is appropriate from professional perspective and whether a term is suitable under a specific category) and content checking (whether the definitions or synonyms of a certain term is correct and related). Based on the positive feedback of quality control, we updated and released the terminology online.

### Web service

We built a website for COVID Term, making it available for users to access each updated data version. For each update round, enriched sub branches with abundant information were required, where up to date COVID resources e.g. the lancet coronavirus theme, NIH 2019 novel coronavirus theme, WHO COVID-19 theme, the New England Journal of Medicine COVID-19 theme [37–40], etc. were constantly followed by COVID team to provide most recent terminology. The terminology towards COVID-19 was named as COVID Term. Earlier versions were also released on the PHDA (Population Health Data Archive) [41].

## Results

### COVID term overview

We constructed a 6-level bilingual terminology system for COVID-19 involving 464 concepts, 724 Chinese terms, 887 English terms, 464 Chinese preferred terms, 464 English preferred terms, 260 Non-preferred Chinese terms, 423 Non-preferred English terms, 42 Chinese definitions, and 5 English definitions. The first-level category was designed as disease, living organism, clinical manifestation, diagnosis and treatment technique, qualifiers, demographic and socioeconomic characteristics, epidemic prevention and control, medical equipment, instruments and materials, psychological assistance, and anatomic site. Detailed term statistics were shown in Table 1.

**Table 1:** The statistics in COVID Term

Category	Concept	Chinese term	English term	Preferred Chinese term	Preferred English term	Chinese synonym	English synonym	Chinese definition	English definition
Disease	52	118	149	52	52	66	97	4	2
Living organism	45	67	71	45	45	22	26	4	0
Clinical manifestation	94	193	219	94	94	99	125	5	0
Diagnosis and treatment technique	83	108	156	83	83	25	73	2	0
Qualifiers	27	31	33	27	27	4	6	15	0
Demographic and socioeconomic characteristics	9	16	12	9	9	7	3	1	2
Epidemic prevention and control	64	74	106	64	64	10	42	7	1
Medical equipment, instruments and materials	49	61	60	49	49	12	11	0	0
Psychological assistance	33	38	50	33	33	5	17	2	0
Anatomic site	9	19	32	9	9	10	23	2	0
Total (without repeat)	464	724	887	464	464	260	423	42	5

### Concept distribution

**Fig. 3:**
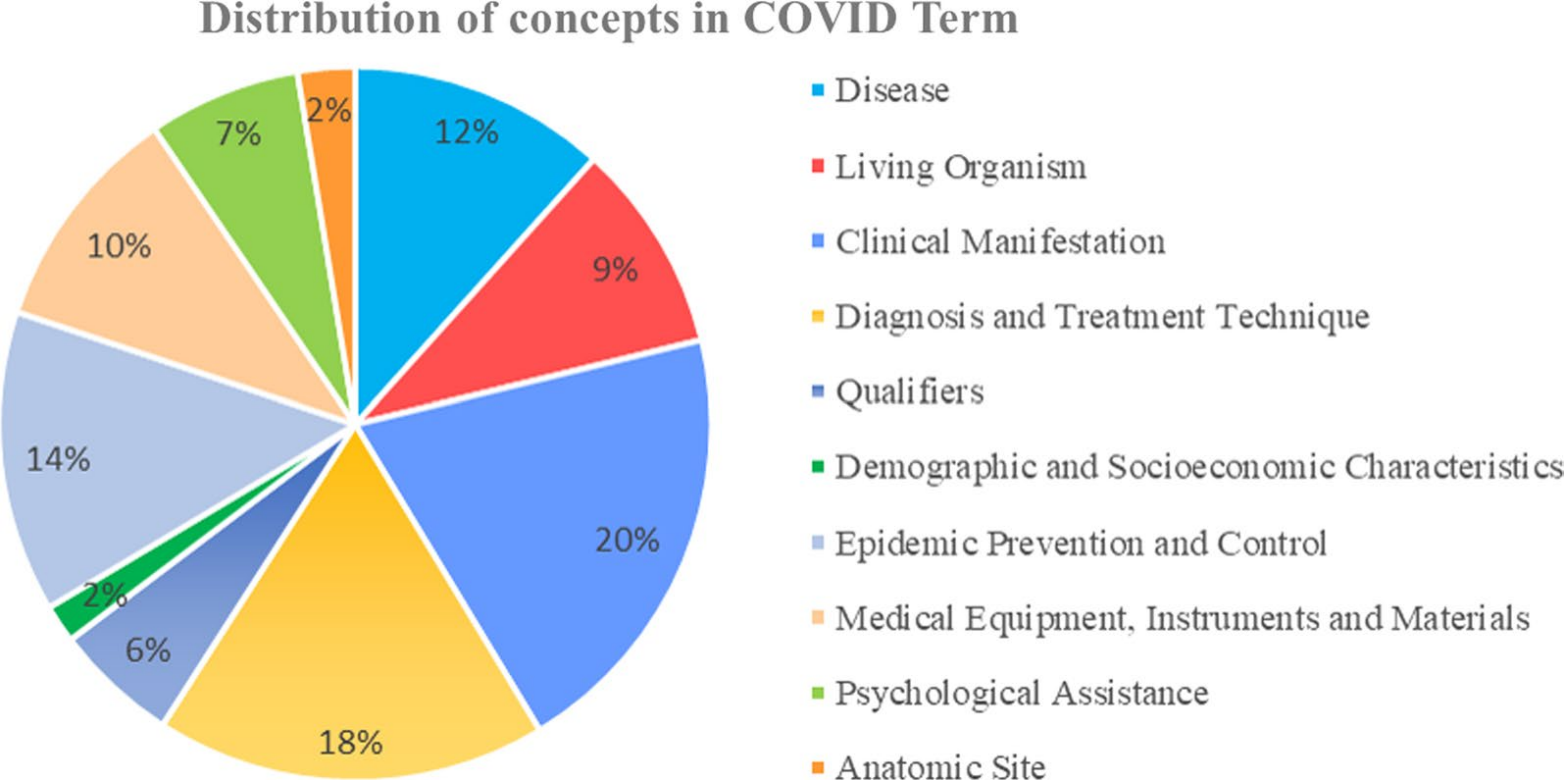
Concept distribution of COVID Term

In this 6-level structure, all first-level terms were root nodes with leafs. There were 10 first-level terms, same as first-level categories, 104 second-level terms, 180 third-level terms, 138 fourth-level terms, 31 fifth-level terms, and 9 sixth-level terms. Sixth-level leaf nodes only existed in category ‘Disease’, ‘Living Organism’, and ‘Anatomic Site’. One example was ‘Living Organism—pathogen—virus—coronavirus—human coronaviruses—severe acute respiratory syndrome coronavirus 2’. Concept distribution in different categories was calculated. Concepts in ‘Clinical Manifestation’, ‘Diagnosis and Treatment Technique’, ‘Epidemic Prevention and Control’ ranked top 3, which accounted for over half concepts, with proportion respectively 20%, 18%, 14%. Concept distribution was shown in Fig. 3.

### Terminology property analysis

**Fig. 4:**
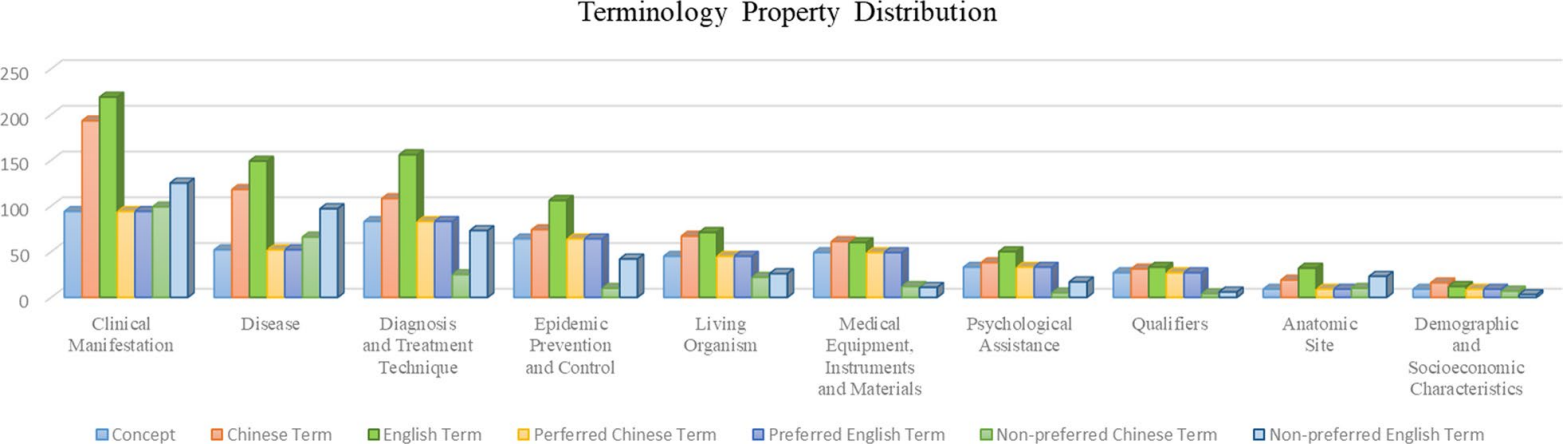
Term property distribution of COVID Term

We performed data statistics on each property of every category including concept, bilingual term count, bilingual synonym count, and bilingual definition count in descending order of concept. Concepts in category ‘Clinical Manifestation’ ranked first with 94 concepts, together with 99 Chinese synonyms and 125 English synonyms. Most English terms took a major proportion in all properties among different categories. Few English definitions were included in the system. Category ‘Qualifiers’ showed the most definitions as 15. Categories with English synonyms over 50 were ‘Clinical Manifestation’, ‘Disease’, and ‘Diagnosis and Treatment Technique’ and, as shown in Fig. 4.

### Semantic type distribution

**Fig. 5:**
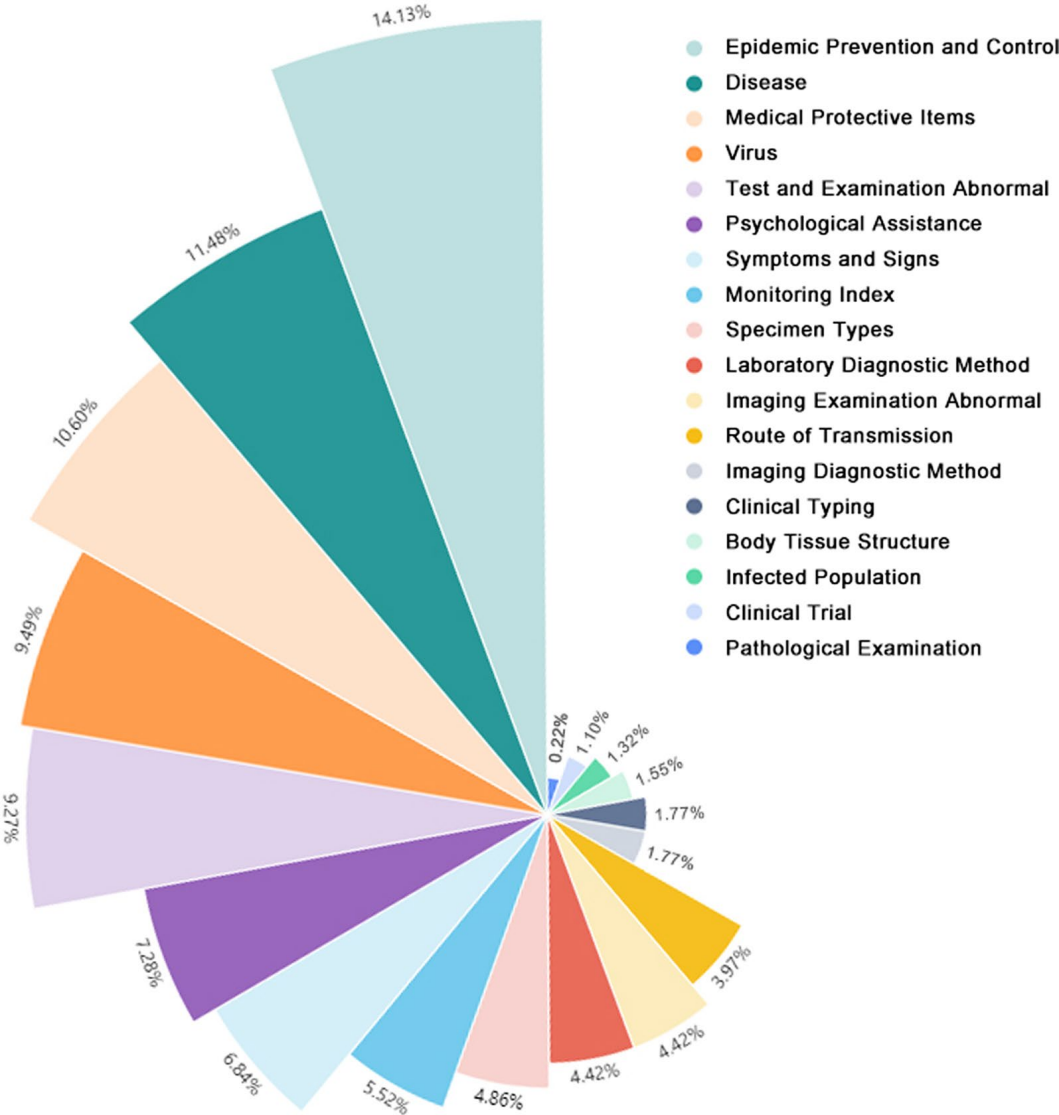
Semantic type distribution of COVID Term

We assigned a semantic type for most of the concepts, which mostly were the top second level term. For example, the semantic type of either latex gloves or gloves was labelled ‘Medical Protective Items’, representing its semantic meaning. There were 18 semantic types in total as illustrated. As seen in Fig. 5, the top 3 semantic types were ‘Epidemic Prevention and Control’, ‘Disease’, and ‘Medical Protective Items’. Concepts in top half semantic types covered nearly 80% of the whole terms. There was only one terminology in pathological examination i.e. biopsy. Different clinical stages were classified into the semantic type ‘Clinical Trial’.

### Website demonstration

**Fig. 6:**
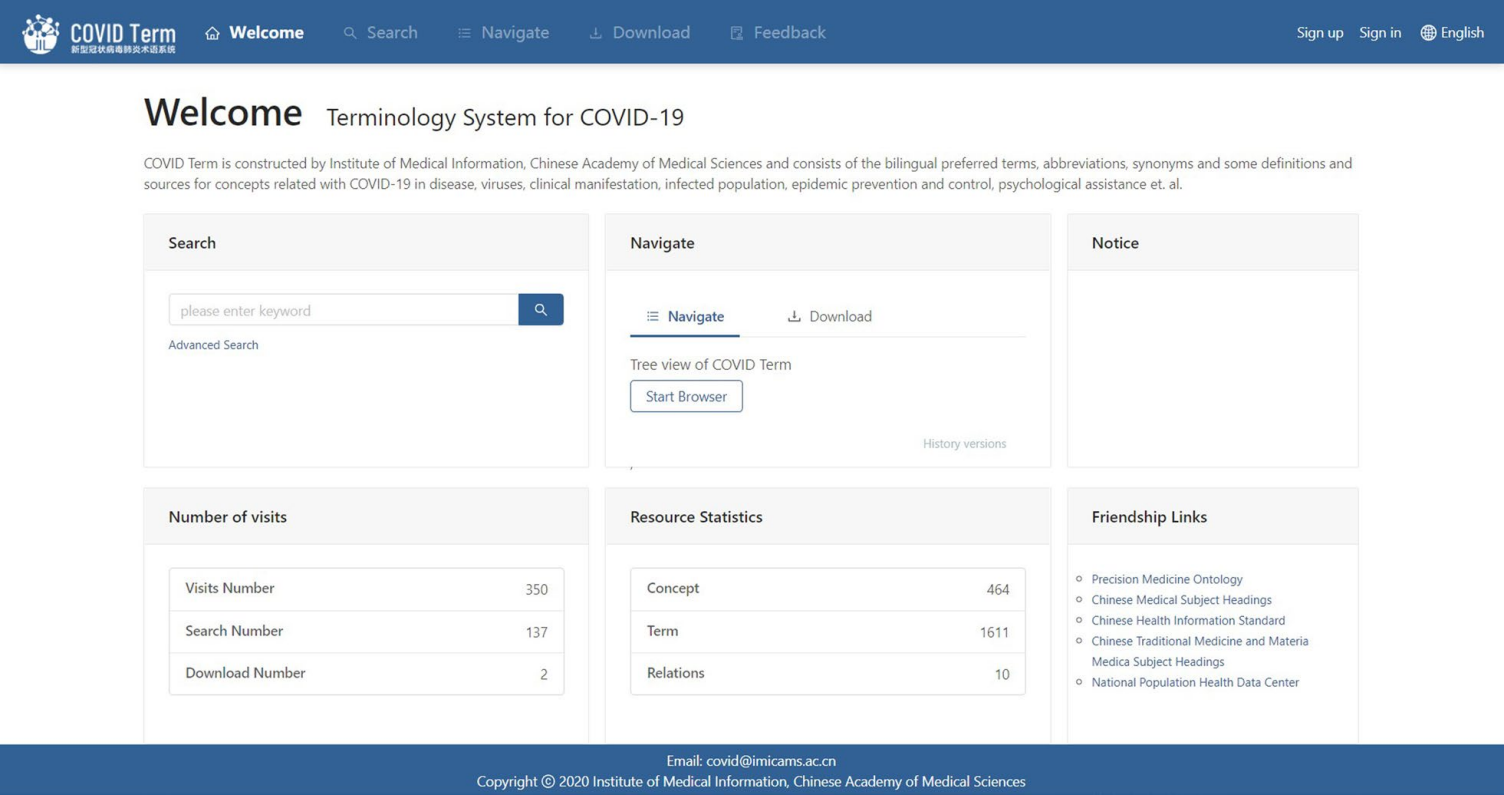
COVID Term website homepage

We built a website for users to access COVID Term, which provided browsing, searching, and downloading, etc. services. On the homepage, users could choose to search, navigate (browse), download, and leave feedback online. Users could also see the visit number statistics for different services. Numbers of concept, term, and relationship type were shown on the same page. The website also provided Friendship Links including ‘Precision Medicine Ontology’, ‘Chinese Medical Subject Headings’, etc. (Fig. 6)

**Fig. 7:**
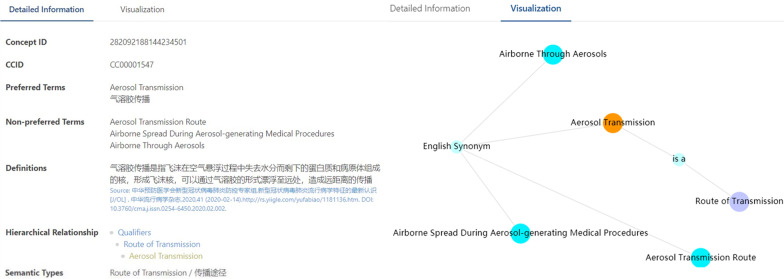
Concept details in COVID Term

In the ‘search’ function, users could search wanted terms through general or advanced search, where searching in different language terms, definitions, exact matches between term and keyword, etc. were available. On the navigation page, users could select each level concept and their subconcepts, details were provided on the right area with concept ID, preferred terms, non-preferred terms, definitions, hierarchical relationship, and semantic types. A simple knowledge graph was also shown in the visualization branch, illustrating the relationship of each concept and related terms, as shown in Fig. 7. Users could download the whole dataset in Excel, XML, JSON format.

## Discussion

### Principle findings

COVID-19, an extremely destructive disease confirmed for several months, has caused over 100 thousand deaths, and more than 1.5 million confirmed cases [5]. This emergency leads to a common scenario that many patients in lack of medical resources are suffering from the disease. With the constant enlargement of the epidemic coverage, related data and information in various types have been growing exponentially. Researchers who did their survey on COVID-related knowledge have strong requirements for information retrieval in different languages, as a result, we created this bilingual terminology system-COVID Term. We referred to authoritative terminology system e.g. ICD, SNOMED CT for their structure design. However, there is obvious differences between COVID Term and them. ICD is focused on diseases which brings benefit for statistical analysis in classification. SNOMED CT is focused on clinical field, which is aiming at electronic exchange of clinical health information and interoperability. Both ICD and SNOMED CT are designed for general medical field with different specific purposes. For a specific disease, more exquisite concept granularity is needed. Consequently, ontology aiming at drug repurposing towards COVID-19 were constructed. COVID Term is a bilingual terminology focused on only COVID-related terms, meeting multilingual retrieval requirements in this specific disease and providing parallel bilingual data for machine algorithms and applications such as information extraction.

We constructed a 6-level 10-category COVID-focused terminology system named COVID Term by collecting and integrating highly related bilingual concepts, synonyms, definitions, and semantic types. 464 bilingual preferred terms, 724 Chinese terms, 887 English terms were included in the terminology system, where each term was classified into a category involving Diagnosis and Treatment Technique, Epidemic Prevention and Control, Psychological Assistance, Clinical Manifestation, Qualifiers, Disease, Anatomic Site, Living Organism, Medical Equipment, Instruments and Materials, and Demographic and Socioeconomic Characteristics. In addition, we provided simple knowledge graphs to illustrate their relationship and built a website so that terminology dataset could be open accessible to all users.

### Application prospects

COVID Term is a terminology dataset applicable for people from all over the world, despite careers, ages, and nationalities, which can be used in many applications and senarios. For people with the cross-lingual requirements, it would support knowledge dissemination and promote scientific achievement sharing towards COVID-19. Furthermore, front-line physicians, clinicians, and other clinical staff could directly look up to the Term as a dictionary, quickly obtaining appropriate ways of specific professional vocabulary aiming at COVID-19 and its definition in different languages. With the continuously increasing demands for scientific discovery of COVID-19, retrieval of high recall and high precision is supposed to be supported for information accessing [42]. COVID Term plays a critical role in this part since all the preferred terms, synonyms, bilingual counterparts and related definitions can be enriched and expanded to the necessary query schemas when searching for literatures. In this way, more scientific result, publishment, and achievement can be widely accessible to users in need, especially for those who desire to know information from cross countries (e.g. USA and China). COVID Term can also be viewed as a cross-lingual database or dataset, the requirement for which has always been proposed to reduce language ambiguity and realize knowledge transmission [43]. For instance, it is a specific parallel corpus prepared for machine translation algorithm and application.

For people with natural language processing requirements, COVID Term could be used in multiple conditions especially via NLP techniques. In one case, when performing data mining in COVID-related medical data, clinical electronic health record is a solid and important choice for its abundant information. However, it could only be automatically used when the unstructured text data are transformed to the structured data, COVID Term could be used for information extraction and transformation. In another case, the publisher, data broker, scientific researchers can use COVID Term in the automatic indexing or annotation of medical documents e.g. biomedical literature, which is also known as coding and is pervasively applied in influential terminology or ontology systems such as ICD, SNOMED CT, UMLS, etc. Name entity recognition could be carried out using the data already labelled in COVID Term for not only electronic health records but also more other medical information resources. Also, COVID Term can provide support for those COVID-related medical documents requiring classification management.

For people with knowledge organization and ontology system construction requirements, we could take maximum advantage of COVID Term in different operations settings and scenarios. Medical informatics scientists, analysts, and other data processing technicians could construct COVID ontology by reusing COVID Term directly, thus providing functions such as data integration and data standardization [44]. Most existed terminology or ontology system (e.g. SNOMED CT, ICD) are aiming at general medical field thus might lack information of this newly-occurred disease. COVID Term could be a supplement for these types of systems with multilingual service. Since the data and properties could be seen as the relationship in COVID Term, more comprehensive knowledge graph construction could be completed via the integration of existing simple small-scale knowledge graphs, where automatic question and answering system for public could be supported by the knowledge network. For instance, if the question is proposed as “what image tests do I need to take for COVID-19?” the answer should involve “Chest imaging”, “chest X-ray” or other imaging diagnostic methods in COVID Term”. In addition, the knowledge graph can also provide assistance of knowledge reasoning and decision making for clinicians, (e.g. inferring a potential disease given existed symptoms).

### Limitations and future studies

Currently, there are only 42 Chinese definitions and 5 English definitions included in the system. More definitions need to be integrated. With the growing data generated from all over the world, more classifications should be included and more relationships are supposed to be designed. For future work, we would continue collecting related terms including the gene terms, expression terms, pathway terms, constructing more mature systems, and designing more interfaces applicable to various platforms. Also, we plan to integrate our data according to the mapping criteria and format of OBO Foundry [45] and submit the data as a complement to the existed systems.

## Conclusions

To promote the COVID-19 related bilingual information retrieval, information dissemination, data reuse through intelligent techniques, we constructed a COVID focused terminology system at vocabulary level in the name of COVID Term. COVID Term is a bilingual terminology system including 464 bilingual COVID related concepts, each with bilingual preferred term, concept ID, and other alternative properties such as bilingual synonyms, bilingual definitions, and semantic type. All terms were accessible through the website, which provided search, navigate, browse, download, and visualization services, etc. COVID Term would promote knowledge dissemination and contribute to advanced intelligent techniques applications.

## Data Availability

The datasets generated and analysed during the current study are available [http://covidterm.imicams.ac.cn].

## Abbreviations

SNOMED CT: Systematized Nomenclature of Medicine–Clinical Terms
UMLS: Unified Medical Language System
NIH: National Institutes of Health
XML: Extensible Markup Language
JSON: JavaScript Object Notation
NLP: Natural Language Processing
